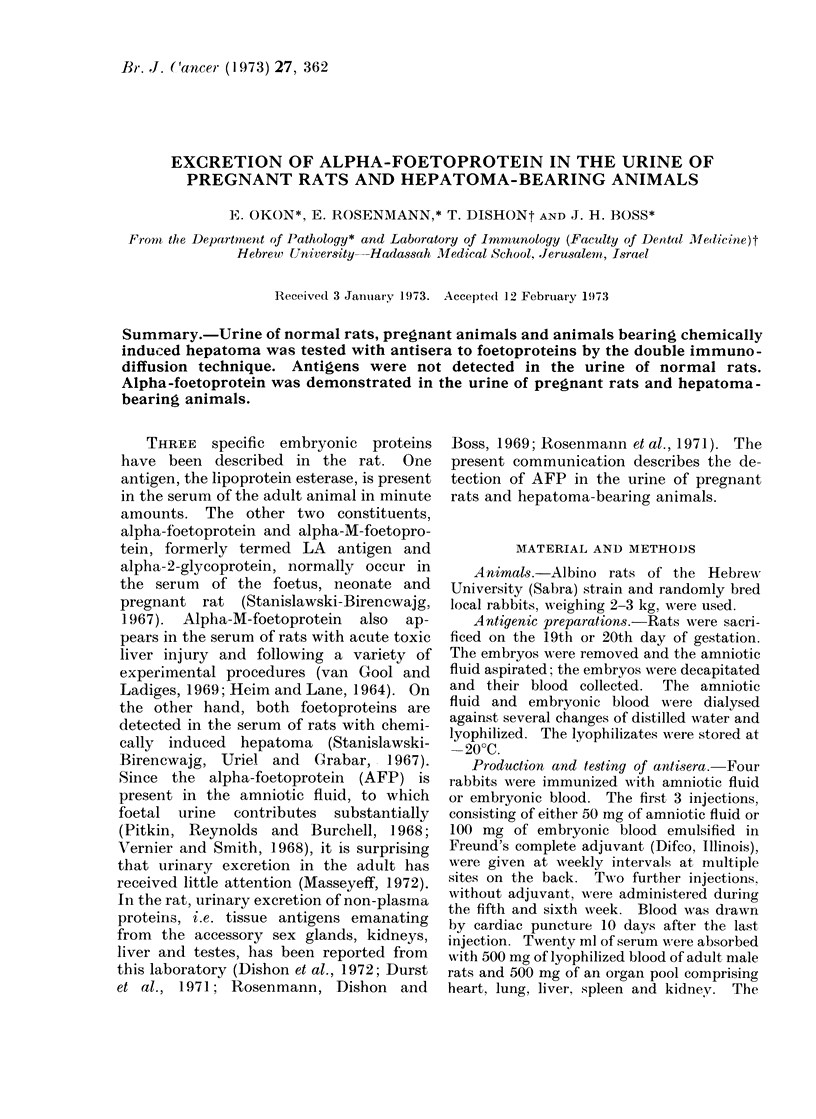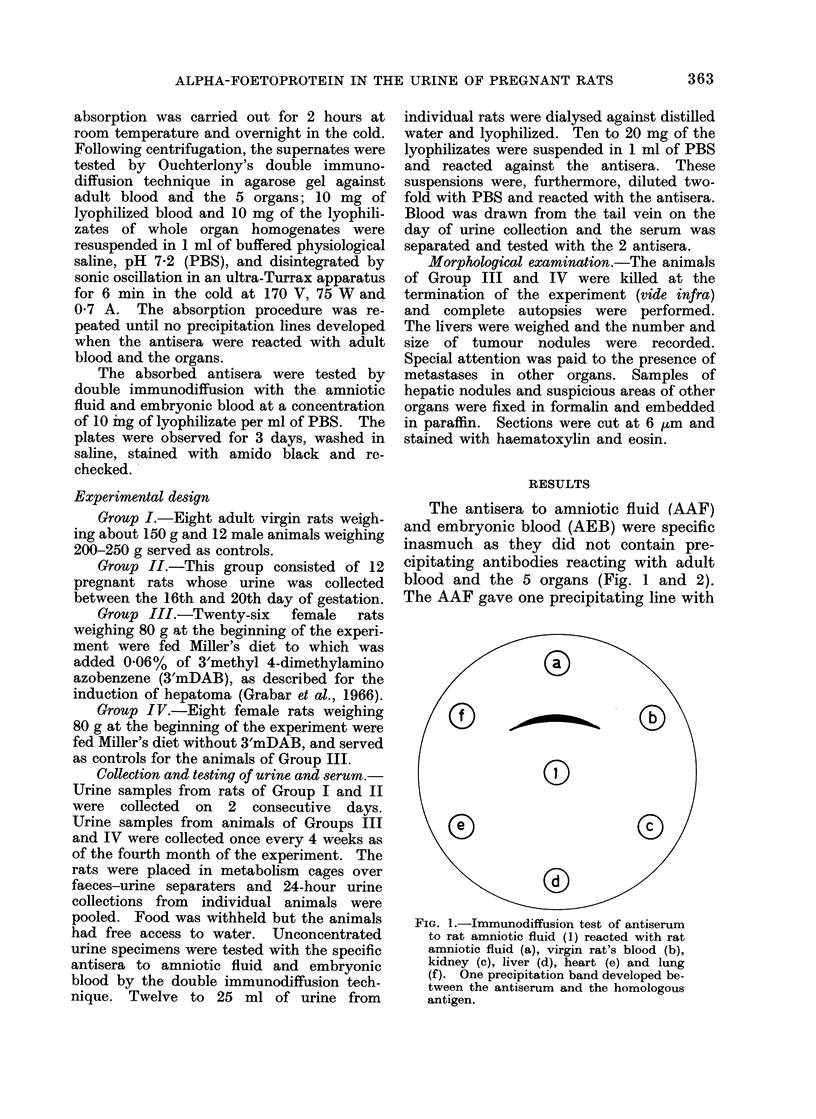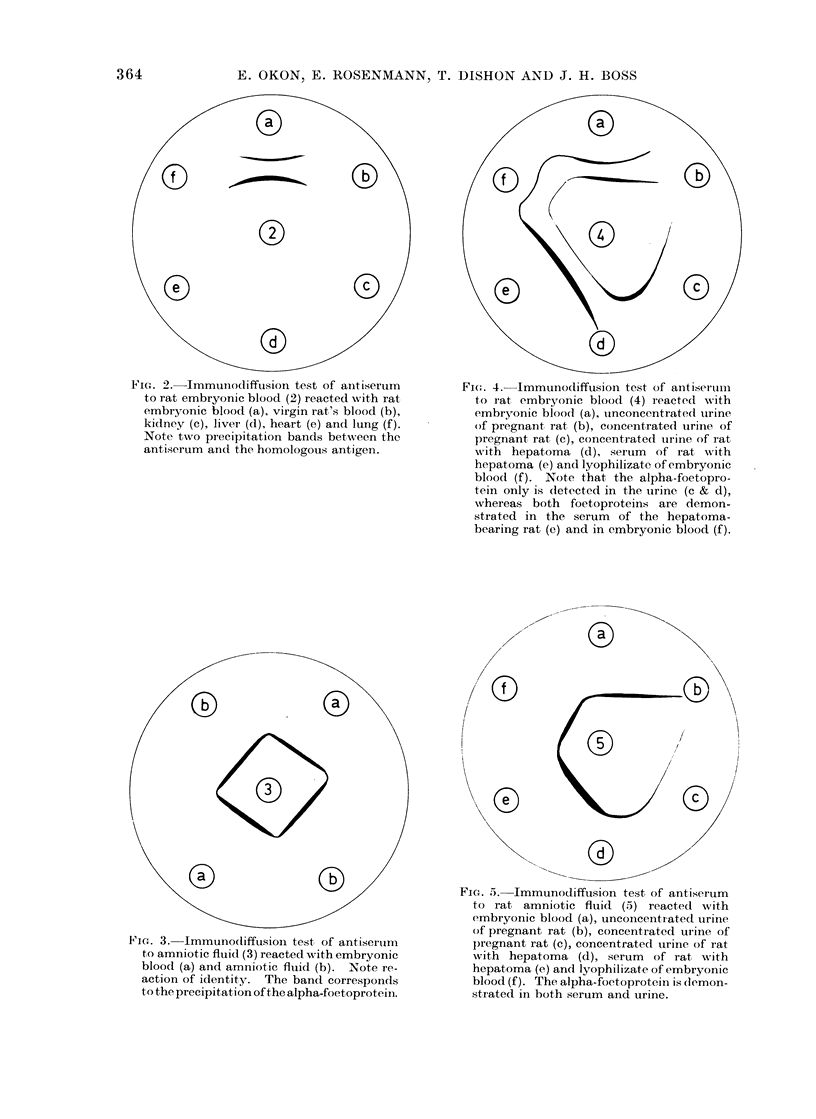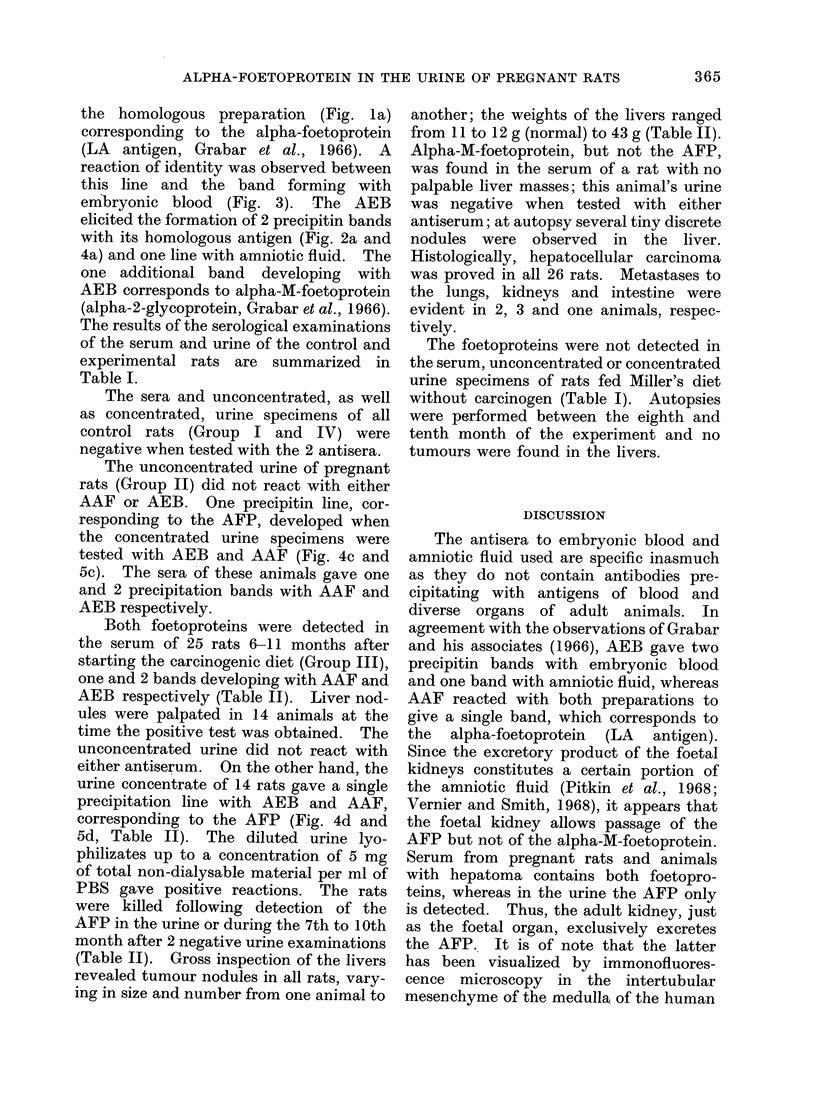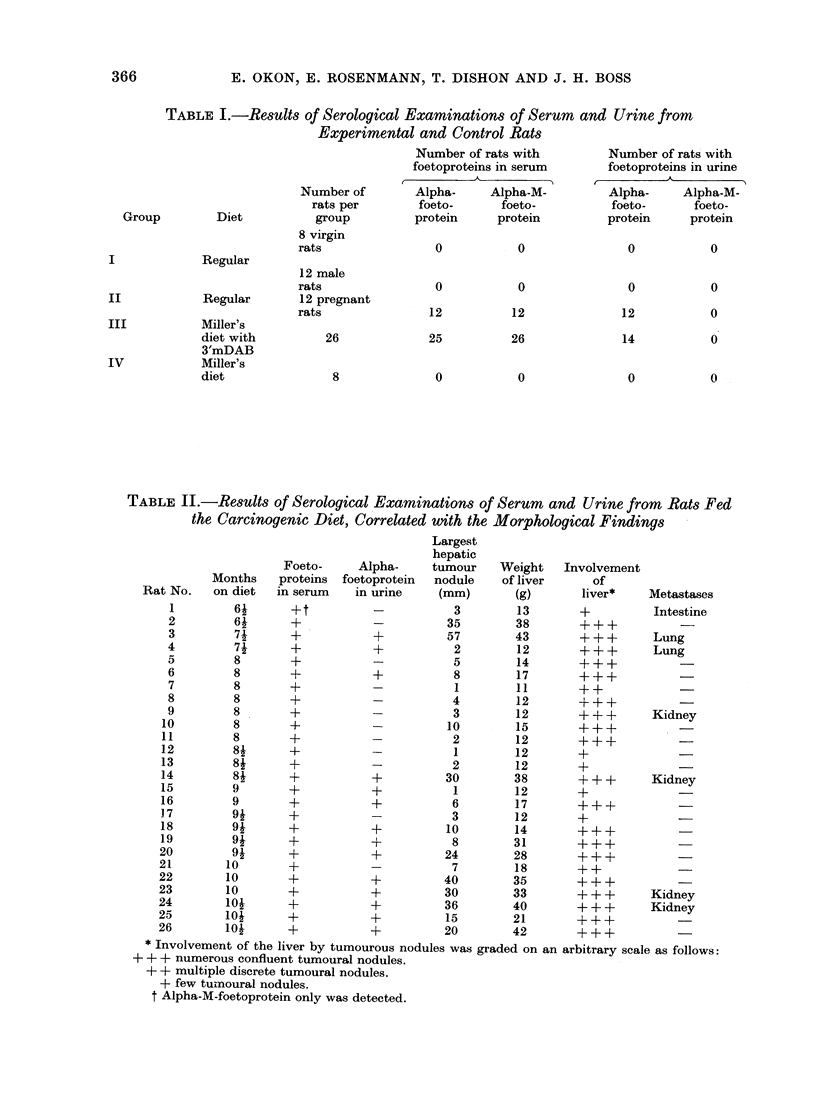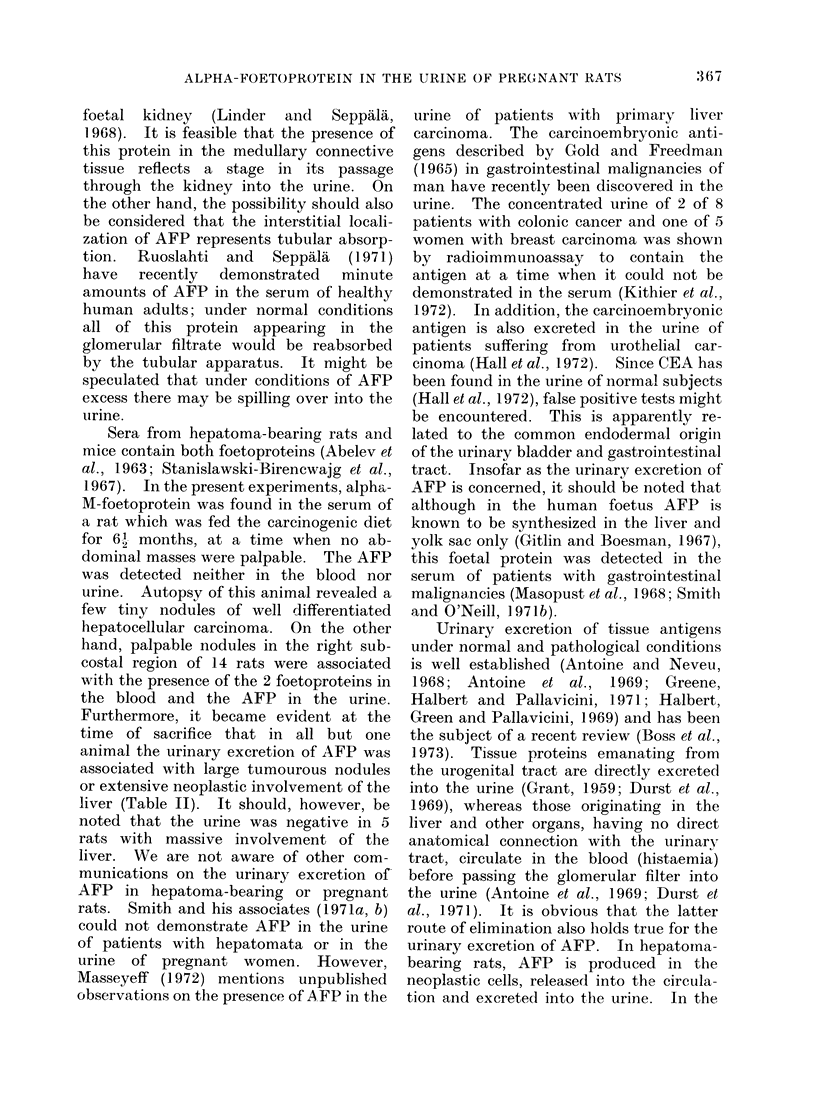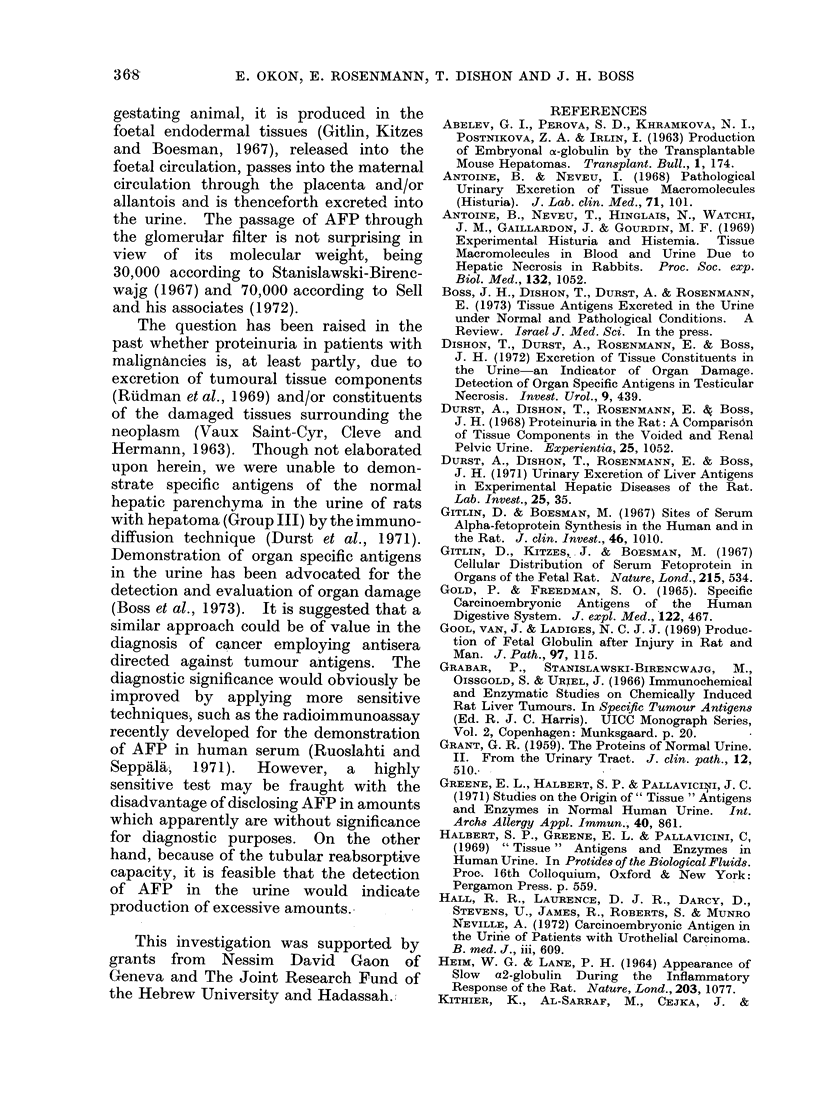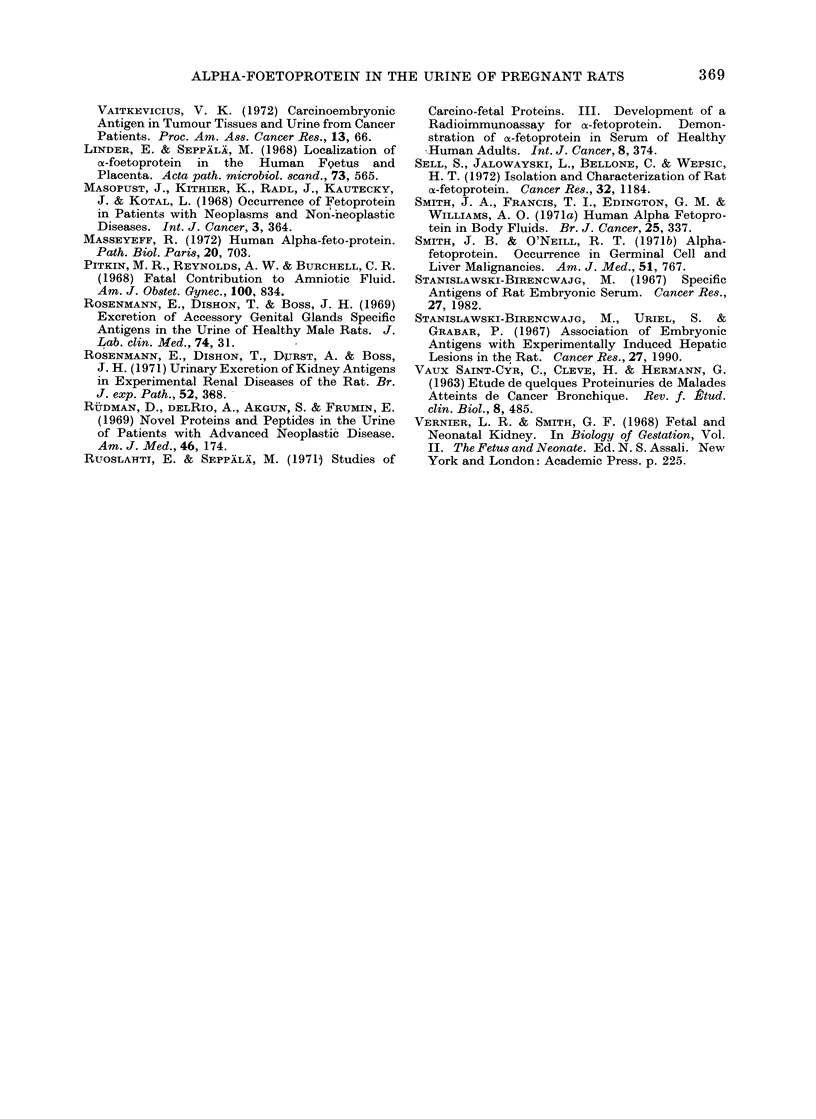# Excretion of Alpha-foetoprotein in the Urine of Pregnant Rats and Hepatoma-bearing animals

**DOI:** 10.1038/bjc.1973.44

**Published:** 1973-05

**Authors:** E. Okon, E. Rosenmann, T. Dishon, J. H. Boss

## Abstract

Urine of normal rats, pregnant animals and animals bearing chemically induced hepatoma was tested with antisera to foetoproteins by the double immunodiffusion technique. Antigens were not detected in the urine of normal rats. Alpha-foetoprotein was demonstrated in the urine of pregnant rats and hepatomabearing animals.


Br. J. Cancer (1973) 27, 362

### EXCRETION OF ALPHA-FOETOPROTEIN IN THE URINE OF PREGNANT RATS AND HEPATOMA-BEARING ANIMALS

E. OKON*, E. ROSENMANN,* T. DISHON† AND J. H. BOSS*

*From the Department of Pathology* and Laboratory of Immunology (Faculty of Dental Medicine)† Hebrew University—Hadassah Medical School, Jerusalem, Israel*

Received 3 January 1973. Accepted 12 February 1973

**Summary.—Urine of normal rats, pregnant animals and animals bearing chemically induced hepatoma was tested with antisera to foetoproteins by the double immunodiffusion technique. Antigens were not detected in the urine of normal rats. Alpha-foetoprotein was demonstrated in the urine of pregnant rats and hepatoma-bearing animals.**

THREE specific embryonic proteins have been described in the rat. One antigen, the lipoprotein esterase, is present in the serum of the adult animal in minute amounts. The other two constituents, alpha-foetoprotein and alpha-M-foetoprotein, formerly termed LA antigen and alpha-2-glycoprotein, normally occur in the serum of the foetus, neonate and pregnant rat (Stanislawski-Birencwajg, 1967). Alpha-M-foetoprotein also appears in the serum of rats with acute toxic liver injury and following a variety of experimental procedures (van Gool and Ladiges, 1969; Heim and Lane, 1964). On the other hand, both foetoproteins are detected in the serum of rats with chemically induced hepatoma (Stanislawski-Birencwajg, Uriel and Grabar, 1967). Since the alpha-foetoprotein (AFP) is present in the amniotic fluid, to which foetal urine contributes substantially (Pitkin, Reynolds and Burchell, 1968; Vernier and Smith, 1968), it is surprising that urinary excretion in the adult has received little attention (Masseyeff, 1972). In the rat, urinary excretion of non-plasma proteins, *i.e.* tissue antigens emanating from the accessory sex glands, kidneys, liver and testes, has been reported from this laboratory (Dishon *et al.*, 1972; Durst *et al.*, 1971; Rosenmann, Dishon and Boss, 1969; Rosenmann *et al.*, 1971). The present communication describes the detection of AFP in the urine of pregnant rats and hepatoma-bearing animals.

### MATERIAL AND METHODS

*Animals.*—Albino rats of the Hebrew University (Sabra) strain and randomly bred local rabbits, weighing 2–3 kg, were used.

*Antigenic preparations.*—Rats were sacrificed on the 19th or 20th day of gestation. The embryos were removed and the amniotic fluid aspirated; the embryos were decapitated and their blood collected. The amniotic fluid and embryonic blood were dialysed against several changes of distilled water and lyophilized. The lyophilizates were stored at −20°C.

*Production and testing of antisera.*—Four rabbits were immunized with amniotic fluid or embryonic blood. The first 3 injections, consisting of either 50 mg of amniotic fluid or 100 mg of embryonic blood emulsified in Freund's complete adjuvant (Difco, Illinois), were given at weekly intervals at multiple sites on the back. Two further injections, without adjuvant, were administered during the fifth and sixth week. Blood was drawn by cardiac puncture 10 days after the last injection. Twenty ml of serum were absorbed with 500 mg of lyophilized blood of adult male rats and 500 mg of an organ pool comprising heart, lung, liver, spleen and kidney. The absorption was carried out for 2 hours at room temperature and overnight in the cold. Following centrifugation, the supernates were tested by Ouchterlony's double immunodiffusion technique in agarose gel against adult blood and the 5 organs; 10 mg of lyophilized blood and 10 mg of the lyophilizates of whole organ homogenates were resuspended in 1 ml of buffered physiological saline, pH 7·2 (PBS), and disintegrated by sonic oscillation in an ultra-Turrax apparatus for 6 min in the cold at 170 V, 75 W and 0·7 A. The absorption procedure was repeated until no precipitation lines developed when the antisera were reacted with adult blood and the organs.

The absorbed antisera were tested by double immunodiffusion with the amniotic fluid and embryonic blood at a concentration of 10 mg of lyophilizate per ml of PBS. The plates were observed for 3 days, washed in saline, stained with amido black and rechecked.

*Experimental design*

*Group I.*—Eight adult virgin rats weighing about 150 g and 12 male animals weighing 200–250 g served as controls.

*Group II.*—This group consisted of 12 pregnant rats whose urine was collected between the 16th and 20th day of gestation.

*Group III.*—Twenty-six female rats weighing 80 g at the beginning of the experiment were fed Miller's diet to which was added 0·06% of 3′methyl 4-dimethylamino azobenzene (3′mDAB), as described for the induction of hepatoma (Grabar *et al.*, 1966).

*Group IV.*—Eight female rats weighing 80 g at the beginning of the experiment were fed Miller's diet without 3′mDAB, and served as controls for the animals of Group III.

*Collection and testing of urine and serum.*—Urine samples from rats of Group I and II were collected on 2 consecutive days. Urine samples from animals of Groups III and IV were collected once every 4 weeks as of the fourth month of the experiment. The rats were placed in metabolism cages over faeces–urine separaters and 24-hour urine collections from individual animals were pooled. Food was withheld but the animals had free access to water. Unconcentrated urine specimens were tested with the specific antisera to amniotic fluid and embryonic blood by the double immunodiffusion technique. Twelve to 25 ml of urine from individual rats were dialysed against distilled water and lyophilized. Ten to 20 mg of the lyophilizates were suspended in 1 ml of PBS and reacted against the antisera. These suspensions were, furthermore, diluted twofold with PBS and reacted with the antisera. Blood was drawn from the tail vein on the day of urine collection and the serum was separated and tested with the 2 antisera.

*Morphological examination.*—The animals of Group III and IV were killed at the termination of the experiment (*vide infra*) and complete autopsies were performed. The livers were weighed and the number and size of tumour nodules were recorded. Special attention was paid to the presence of metastases in other organs. Samples of hepatic nodules and suspicious areas of other organs were fixed in formalin and embedded in paraffin. Sections were cut at 6 μm and stained with haematoxylin and eosin.

### RESULTS

The antisera to amniotic fluid (AAF) and embryonic blood (AEB) were specific inasmuch as they did not contain precipitating antibodies reacting with adult blood and the 5 organs (Fig. 1 and 2). The AAF gave one precipitating line with

FIG. 1.—Immunodiffusion test of antiserum to rat amniotic fluid (1) reacted with rat amniotic fluid (a), virgin rat's blood (b), kidney (c), liver (d), heart (e) and lung (f). One precipitation band developed between the antiserum and the homologous antigen.

FIG. 2.—Immunodiffusion test of antiserum to rat embryonic blood (2) reacted with rat embryonic blood (a), virgin rat's blood (b), kidney (c), liver (d), heart (e) and lung (f). Note two precipitation bands between the antiserum and the homologous antigen.

FIG. 4.—Immunodiffusion test of antiserum to rat embryonic blood (4) reacted with embryonic blood (a), unconcentrated urine of pregnant rat (b), concentrated urine of pregnant rat (c), concentrated urine of rat with hepatoma (d), serum of rat with hepatoma (e) and lyophilizate of embryonic blood (f). Note that the alpha-foetoprotein only is detected in the urine (c & d), whereas both foetoproteins are demonstrated in the serum of the hepatoma-bearing rat (e) and in embryonic blood (f).

FIG. 3.—Immunodiffusion test of antiserum to amniotic fluid (3) reacted with embryonic blood (a) and amniotic fluid (b). Note reaction of identity. The band corresponds to the precipitation of the alpha-foetoprotein.

FIG. 5.—Immunodiffusion test of antiserum to rat amniotic fluid (5) reacted with embryonic blood (a), unconcentrated urine of pregnant rat (b), concentrated urine of rat with hepatoma (d), serum of rat with hepatoma (e) and lyophilizate of embryonic blood (f). The alpha-foetoprotein is demonstrated in both serum and urine.

the homologous preparation (Fig. 1a) corresponding to the alpha-foetoprotein (LA antigen, Grabar *et al.*, 1966). A reaction of identity was observed between this line and the band forming with embryonic blood (Fig. 3). The AEB elicited the formation of 2 precipitin bands with its homologous antigen (Fig. 2a and 4a) and one line with amniotic fluid. The one additional band developing with AEB corresponds to alpha-M-foetoprotein (alpha-2-glycoprotein, Grabar *et al.*, 1966). The results of the serological examinations of the serum and urine of the control and experimental rats are summarized in Table I.

The sera and unconcentrated, as well as concentrated, urine specimens of all control rats (Group I and IV) were negative when tested with the 2 antisera.

The unconcentrated urine of pregnant rats (Group II) did not react with either AAF or AEB. One precipitin line, corresponding to the AFP, developed when the concentrated urine specimens were tested with AEB and AAF (Fig. 4c and 5c). The sera of these animals gave one and 2 precipitation bands with AAF and AEB respectively.

Both foetoproteins were detected in the serum of 25 rats 6–11 months after starting the carcinogenic diet (Group III), one and 2 bands developing with AAF and AEB respectively (Table II). Liver nodules were palpated in 14 animals at the time the positive test was obtained. The unconcentrated urine did not react with either antiserum. On the other hand, the urine concentrate of 14 rats gave a single precipitation line with AEB and AAF, corresponding to the AFP (Fig. 4d and 5d, Table II). The diluted urine lyophilizates up to a concentration of 5 mg of total non-dialysable material per ml of PBS gave positive reactions. The rats were killed following detection of the AFP in the urine or during the 7th to 10th month after 2 negative urine examinations (Table II). Gross inspection of the livers revealed tumour nodules in all rats, varying in size and number from one animal to another; the weights of the livers ranged from 11 to 12 g (normal) to 43 g (Table II). Alpha-M-foetoprotein, but not the AFP, was found in the serum of a rat with no palpable liver masses; this animal's urine was negative when tested with either antiserum; at autopsy several tiny discrete nodules were observed in the liver. Histologically, hepatocellular carcinoma was proved in all 26 rats. Metastases to the lungs, kidneys and intestine were evident in 2, 3 and one animals, respectively.

The foetoproteins were not detected in the serum, unconcentrated or concentrated urine specimens of rats fed Miller's diet without carcinogen (Table I). Autopsies were performed between the eighth and tenth month of the experiment and no tumours were found in the livers.

### DISCUSSION

The antisera to embryonic blood and amniotic fluid used are specific inasmuch as they do not contain antibodies precipitating with antigens of blood and diverse organs of adult animals. In agreement with the observations of Grabar and his associates (1966), AEB gave two precipitin bands with embryonic blood and one band with amniotic fluid, whereas AAF reacted with both preparations to give a single band, which corresponds to the alpha-foetoprotein (LA antigen). Since the excretory product of the foetal kidneys constitutes a certain portion of the amniotic fluid (Pitkin *et al.*, 1968; Vernier and Smith, 1968), it appears that the foetal kidney allows passage of the AFP but not of the alpha-M-foetoprotein. Serum from pregnant rats and animals with hepatoma contains both foetoproteins, whereas in the urine the AFP only is detected. Thus, the adult kidney, just as the foetal organ, exclusively excretes the AFP. It is of note that the latter has been visualized by immonofluorescence microscopy in the intertubular mesenchyme of the medulla of the human

TABLE I.—*Results of Serological Examinations of Serum and Urine from Experimental and Control Rats*

| Group | Diet | Number of rats per group | Number of rats with foetoproteins in serum | | Number of rats with foetoproteins in urine | |
|---|---|---|---|---|---|---|
| | | | Alpha-foeto-protein | Alpha-M-foeto-protein | Alpha-foeto-protein | Alpha-M-foeto-protein |
| I | Regular | 8 virgin rats | 0 | 0 | 0 | 0 |
| | | 12 male rats | 0 | 0 | 0 | 0 |
| II | Regular | 12 pregnant rats | 12 | 12 | 12 | 0 |
| III | Miller's diet with 3′mDAB | 26 | 25 | 26 | 14 | 0 |
| IV | Miller's diet | 8 | 0 | 0 | 0 | 0 |

TABLE II.—*Results of Serological Examinations of Serum and Urine from Rats Fed the Carcinogenic Diet, Correlated with the Morphological Findings*

| Rat No. | Months on diet | Foeto-proteins in serum | Alpha-foetoprotein in urine | Largest hepatic tumour nodule (mm) | Weight of liver (g) | Involvement of liver* | Metastases |
|---|---|---|---|---|---|---|---|
| 1 | 6½ | +† | — | 3 | 13 | + | Intestine |
| 2 | 6½ | + | — | 35 | 38 | +++ | — |
| 3 | 7½ | + | + | 57 | 43 | +++ | Lung |
| 4 | 7½ | + | + | 2 | 12 | +++ | Lung |
| 5 | 8 | + | — | 5 | 14 | +++ | — |
| 6 | 8 | + | + | 8 | 17 | +++ | — |
| 7 | 8 | + | — | 1 | 11 | ++ | — |
| 8 | 8 | + | — | 4 | 12 | +++ | — |
| 9 | 8 | + | — | 3 | 12 | +++ | Kidney |
| 10 | 8 | + | — | 10 | 15 | +++ | — |
| 11 | 8 | + | — | 2 | 12 | +++ | — |
| 12 | 8½ | + | — | 1 | 12 | + | — |
| 13 | 8½ | + | — | 2 | 12 | + | — |
| 14 | 8½ | + | + | 30 | 38 | +++ | Kidney |
| 15 | 9 | + | + | 1 | 12 | + | — |
| 16 | 9 | + | + | 6 | 17 | +++ | — |
| 17 | 9½ | + | — | 3 | 12 | + | — |
| 18 | 9½ | + | + | 10 | 14 | +++ | — |
| 19 | 9½ | + | + | 8 | 31 | +++ | — |
| 20 | 9½ | + | + | 24 | 28 | +++ | — |
| 21 | 10 | + | — | 7 | 18 | ++ | — |
| 22 | 10 | + | + | 40 | 35 | +++ | — |
| 23 | 10 | + | + | 30 | 33 | +++ | Kidney |
| 24 | 10½ | + | + | 36 | 40 | +++ | Kidney |
| 25 | 10½ | + | + | 15 | 21 | +++ | — |
| 26 | 10½ | + | + | 20 | 42 | +++ | — |

* Involvement of the liver by tumourous nodules was graded on an arbitrary scale as follows:
+++ numerous confluent tumoural nodules.
++ multiple discrete tumoural nodules.
+ few tumoural nodules.
† Alpha-M-foetoprotein only was detected.

foetal kidney (Linder and Seppälä, 1968). It is feasible that the presence of this protein in the medullary connective tissue reflects a stage in its passage through the kidney into the urine. On the other hand, the possibility should also be considered that the interstitial localization of AFP represents tubular absorption. Ruoslahti and Seppälä (1971) have recently demonstrated minute amounts of AFP in the serum of healthy human adults; under normal conditions all of this protein appearing in the glomerular filtrate would be reabsorbed by the tubular apparatus. It might be speculated that under conditions of AFP excess there may be spilling over into the urine.

Sera from hepatoma-bearing rats and mice contain both foetoproteins (Abelev *et al.*, 1963; Stanislawski-Birencwajg *et al.*, 1967). In the present experiments, alpha-M-foetoprotein was found in the serum of a rat which was fed the carcinogenic diet for $6\frac{1}{2}$ months, at a time when no abdominal masses were palpable. The AFP was detected neither in the blood nor urine. Autopsy of this animal revealed a few tiny nodules of well differentiated hepatocellular carcinoma. On the other hand, palpable nodules in the right subcostal region of 14 rats were associated with the presence of the 2 foetoproteins in the blood and the AFP in the urine. Furthermore, it became evident at the time of sacrifice that in all but one animal the urinary excretion of AFP was associated with large tumourous nodules or extensive neoplastic involvement of the liver (Table II). It should, however, be noted that the urine was negative in 5 rats with massive involvement of the liver. We are not aware of other communications on the urinary excretion of AFP in hepatoma-bearing or pregnant rats. Smith and his associates (1971*a*, *b*) could not demonstrate AFP in the urine of patients with hepatomata or in the urine of pregnant women. However, Masseyeff (1972) mentions unpublished observations on the presence of AFP in the urine of patients with primary liver carcinoma. The carcinoembryonic antigens described by Gold and Freedman (1965) in gastrointestinal malignancies of man have recently been discovered in the urine. The concentrated urine of 2 of 8 patients with colonic cancer and one of 5 women with breast carcinoma was shown by radioimmunoassay to contain the antigen at a time when it could not be demonstrated in the serum (Kithier *et al.*, 1972). In addition, the carcinoembryonic antigen is also excreted in the urine of patients suffering from urothelial carcinoma (Hall *et al.*, 1972). Since CEA has been found in the urine of normal subjects (Hall *et al.*, 1972), false positive tests might be encountered. This is apparently related to the common endodermal origin of the urinary bladder and gastrointestinal tract. Insofar as the urinary excretion of AFP is concerned, it should be noted that although in the human foetus AFP is known to be synthesized in the liver and yolk sac only (Gitlin and Boesman, 1967), this foetal protein was detected in the serum of patients with gastrointestinal malignancies (Masopust *et al.*, 1968; Smith and O'Neill, 1971*b*).

Urinary excretion of tissue antigens under normal and pathological conditions is well established (Antoine and Neveu, 1968; Antoine *et al.*, 1969; Greene, Halbert and Pallavicini, 1971; Halbert, Green and Pallavicini, 1969) and has been the subject of a recent review (Boss *et al.*, 1973). Tissue proteins emanating from the urogenital tract are directly excreted into the urine (Grant, 1959; Durst *et al.*, 1969), whereas those originating in the liver and other organs, having no direct anatomical connection with the urinary tract, circulate in the blood (histaemia) before passing the glomerular filter into the urine (Antoine *et al.*, 1969; Durst *et al.*, 1971). It is obvious that the latter route of elimination also holds true for the urinary excretion of AFP. In hepatoma-bearing rats, AFP is produced in the neoplastic cells, released into the circulation and excreted into the urine. In the gestating animal, it is produced in the foetal endodermal tissues (Gitlin, Kitzes and Boesman, 1967), released into the foetal circulation, passes into the maternal circulation through the placenta and/or allantois and is thenceforth excreted into the urine. The passage of AFP through the glomerular filter is not surprising in view of its molecular weight, being 30,000 according to Stanislawski-Birencwajg (1967) and 70,000 according to Sell and his associates (1972).

The question has been raised in the past whether proteinuria in patients with malignancies is, at least partly, due to excretion of tumoural tissue components (Rüdman *et al.*, 1969) and/or constituents of the damaged tissues surrounding the neoplasm (Vaux Saint-Cyr, Cleve and Hermann, 1963). Though not elaborated upon herein, we were unable to demonstrate specific antigens of the normal hepatic parenchyma in the urine of rats with hepatoma (Group III) by the immunodiffusion technique (Durst *et al.*, 1971). Demonstration of organ specific antigens in the urine has been advocated for the detection and evaluation of organ damage (Boss *et al.*, 1973). It is suggested that a similar approach could be of value in the diagnosis of cancer employing antisera directed against tumour antigens. The diagnostic significance would obviously be improved by applying more sensitive techniques, such as the radioimmunoassay recently developed for the demonstration of AFP in human serum (Ruoslahti and Seppälä, 1971). However, a highly sensitive test may be fraught with the disadvantage of disclosing AFP in amounts which apparently are without significance for diagnostic purposes. On the other hand, because of the tubular reabsorptive capacity, it is feasible that the detection of AFP in the urine would indicate production of excessive amounts.

This investigation was supported by grants from Nessim David Gaon of Geneva and The Joint Research Fund of the Hebrew University and Hadassah.

REFERENCES

ABELEV, G. I., PEROVA, S. D., KHRAMKOVA, N. I., POSTNIKOVA, Z. A. & IRLIN, I. (1963) Production of Embryonal α-globulin by the Transplantable Mouse Hepatomas. *Transplant. Bull.*, **1**, 174.

ANTOINE, B. & NEVEU, I. (1968) Pathological Urinary Excretion of Tissue Macromolecules (Histuria). *J. Lab. clin. Med.*, **71**, 101.

ANTOINE, B., NEVEU, T., HINGLAIS, N., WATCHI, J. M., GAILLARDON, J. & GOURDIN, M. F. (1969) Experimental Histuria and Histemia. Tissue Macromolecules in Blood and Urine Due to Hepatic Necrosis in Rabbits. *Proc. Soc. exp. Biol. Med.*, **132**, 1052.

BOSS, J. H., DISHON, T., DURST, A. & ROSENMANN, E. (1973) Tissue Antigens Excreted in the Urine under Normal and Pathological Conditions. A Review. *Israel J. Med. Sci.* In the press.

DISHON, T., DURST, A., ROSENMANN, E. & BOSS, J. H. (1972) Excretion of Tissue Constituents in the Urine—an Indicator of Organ Damage. Detection of Organ Specific Antigens in Testicular Necrosis. *Invest. Urol.*, **9**, 439.

DURST, A., DISHON, T., ROSENMANN, E. & BOSS, J. H. (1968) Proteinuria in the Rat: A Comparison of Tissue Components in the Voided and Renal Pelvic Urine. *Experientia*, **25**, 1052.

DURST, A., DISHON, T., ROSENMANN, E. & BOSS, J. H. (1971) Urinary Excretion of Liver Antigens in Experimental Hepatic Diseases of the Rat. *Lab. Invest.*, **25**, 35.

GITLIN, D. & BOESMAN, M. (1967) Sites of Serum Alpha-fetoprotein Synthesis in the Human and in the Rat. *J. clin. Invest.*, **46**, 1010.

GITLIN, D., KITZES, J. & BOESMAN, M. (1967) Cellular Distribution of Serum Fetoprotein in Organs of the Fetal Rat. *Nature, Lond.*, **215**, 534.

GOLD, P. & FREEDMAN, S. O. (1965). Specific Carcinoembryonic Antigens of the Human Digestive System. *J. expl. Med.*, **122**, 467.

GOOL, VAN, J. & LADIGES, N. C. J. J. (1969) Production of Fetal Globulin after Injury in Rat and Man. *J. Path.*, **97**, 115.

GRABAR, P., STANISLAWSKI-BIRENCWAJG, M., OISSGOLD, S. & URIEL, J. (1966) Immunochemical and Enzymatic Studies on Chemically Induced Rat Liver Tumours. In *Specific Tumour Antigens* (Ed. R. J. C. Harris). UICC Monograph Series, Vol. 2, Copenhagen: Munksgaard. p. 20.

GRANT, G. R. (1959). The Proteins of Normal Urine. II. From the Urinary Tract. *J. clin. path.*, **12**, 510.

GREENE, E. L., HALBERT, S. P. & PALLAVICINI, J. C. (1971) Studies on the Origin of "Tissue" Antigens and Enzymes in Normal Human Urine. *Int. Archs Allergy Appl. Immun.*, **40**, 861.

HALBERT, S. P., GREENE, E. L. & PALLAVICINI, C, (1969) "Tissue" Antigens and Enzymes in Human Urine. In *Protides of the Biological Fluids.* Proc. 16th Colloquium, Oxford & New York: Pergamon Press. p. 559.

HALL, R. R., LAURENCE, D. J. R., DARCY, D., STEVENS, U., JAMES, R., ROBERTS, S. & MUNRO NEVILLE, A. (1972) Carcinoembryonic Antigen in the Urine of Patients with Urothelial Carcinoma. *B. med. J.*, iii, 609.

HEIM, W. G. & LANE, P. H. (1964) Appearance of Slow α2-globulin During the Inflammatory Response of the Rat. *Nature, Lond.*, **203**, 1077.

KITHIER, K., AL-SARRAF, M., CEJKA, J. &

VAITKEVICIUS, V. K. (1972) Carcinoembryonic Antigen in Tumour Tissues and Urine from Cancer Patients. *Proc. Am. Ass. Cancer Res.*, **13**, 66.

LINDER, E. & SEPPÄLÄ, M. (1968) Localization of α-foetoprotein in the Human Foetus and Placenta. *Acta path. microbiol. scand.*, **73**, 565.

MASOPUST, J., KITHIER, K., RADL, J., KAUTECKY, J. & KOTAL, L. (1968) Occurrence of Fetoprotein in Patients with Neoplasms and Non-neoplastic Diseases. *Int. J. Cancer*, **3**, 364.

MASSEYEFF, R. (1972) Human Alpha-feto-protein. *Path. Biol. Paris*, **20**, 703.

PITKIN, M. R., REYNOLDS, A. W. & BURCHELL, C. R. (1968) Fatal Contribution to Amniotic Fluid. *Am. J. Obstet. Gynec.*, **100**, 834.

ROSENMANN, E., DISHON, T. & BOSS, J. H. (1969) Excretion of Accessory Genital Glands Specific Antigens in the Urine of Healthy Male Rats. *J. Lab. clin. Med.*, **74**, 31.

ROSENMANN, E., DISHON, T., DURST, A. & BOSS, J. H. (1971) Urinary Excretion of Kidney Antigens in Experimental Renal Diseases of the Rat. *Br. J. exp. Path.*, **52**, 368.

RÜDMAN, D., DELRIO, A., AKGUN, S. & FRUMIN, E. (1969) Novel Proteins and Peptides in the Urine of Patients with Advanced Neoplastic Disease. *Am. J. Med.*, **46**, 174.

RUOSLAHTI, E. & SEPPÄLÄ, M. (1971) Studies of Carcino-fetal Proteins. III. Development of a Radioimmunoassay for α-fetoprotein. Demonstration of α-fetoprotein in Serum of Healthy Human Adults. *Int. J. Cancer*, **8**, 374.

SELL, S., JALOWAYSKI, L., BELLONE, C. & WEPSIC, H. T. (1972) Isolation and Characterization of Rat α-fetoprotein. *Cancer Res.*, **32**, 1184.

SMITH, J. A., FRANCIS, T. I., EDINGTON, G. M. & WILLIAMS, A. O. (1971*a*) Human Alpha Fetoprotein in Body Fluids. *Br. J. Cancer*, **25**, 337.

SMITH, J. B. & O'NEILL, R. T. (1971*b*) Alpha-fetoprotein. Occurrence in Germinal Cell and Liver Malignancies. *Am. J. Med.*, **51**, 767.

STANISLAWSKI-BIRENCWAJG, M. (1967) Specific Antigens of Rat Embryonic Serum. *Cancer Res.*, **27**, 1982.

STANISLAWSKI-BIRENCWAJG, M., URIEL, S. & GRABAR, P. (1967) Association of Embryonic Antigens with Experimentally Induced Hepatic Lesions in the Rat. *Cancer Res.*, **27**, 1990.

VAUX SAINT-CYR, C., CLEVE, H. & HERMANN, G. (1963) Etude de quelques Proteinuries de Malades Atteints de Cancer Bronchique. *Rev. f. Êtud. clin. Biol.*, **8**, 485.

VERNIER, L. R. & SMITH, G. F. (1968) Fetal and Neonatal Kidney. In *Biology of Gestation*, Vol. II. *The Fetus and Neonate*. Ed. N. S. Assali. New York and London: Academic Press. p. 225.